# Viewpoints among experts and the public in the Netherlands on including a lifestyle criterion in the healthcare priority setting

**DOI:** 10.1111/hex.13385

**Published:** 2021-11-29

**Authors:** Charlotte M. Dieteren, Vivian T. Reckers‐Droog, Sara Schrama, Dynothra de Boer, Job van Exel

**Affiliations:** ^1^ Department of Health Economics, Erasmus School of Health Policy and Management Erasmus University Rotterdam Rotterdam The Netherlands; ^2^ Erasmus Centre for Health Economics Rotterdam (EsCHER) Erasmus University Rotterdam Rotterdam The Netherlands

**Keywords:** healthcare, lifestyle, Q methodology, rationing, viewpoints

## Abstract

**Context:**

It remains unclear whether there would be societal support for a lifestyle criterion for the healthcare priority setting. This study examines the viewpoints of experts in healthcare and the public regarding support for a lifestyle‐related decision criterion, relative to support for the currently applied criteria, in the healthcare priority setting in the Netherlands.

**Methods:**

We conducted a Q methodology study in samples of experts in healthcare (*n* = 37) and the public (*n* = 44). Participants (total sample *N* = 81) ranked 34 statements that reflected currently applied decision criteria as well as a lifestyle criterion for setting priorities in healthcare. The ranking data were subjected to principal component analysis, followed by oblimin rotation, to identify clusters of participants with similar viewpoints.

**Findings:**

We identified four viewpoints. Participants with Viewpoint 1 believe that treatments that have been proven to be effective should be reimbursed. Those with Viewpoint 2 believe that life is precious and every effort should be made to save a life, even when treatment still results in a very poor state of health. Those with Viewpoint 3 accept government intervention in unhealthy lifestyles and believe that individual responsibility should be taken into account in reimbursement decisions. Participants with Viewpoint 4 attribute importance to the cost‐effectiveness of treatments; however, when priorities have to be set, treatment effects are considered most important. All viewpoints were supported by a mix of public and experts, but Viewpoint 1 was mostly supported by experts and the other viewpoints were mostly supported by members of the public.

**Conclusions:**

This study identified four distinct viewpoints on the healthcare priority setting in the Netherlands, each supported by a mix of experts and members of the public. There seems to be some, but limited, support for a lifestyle criterion—in particular, among members of the public. Experts seem to favour the decision criteria that are currently applied. The diversity in views deserves attention when policymakers want to adhere to societal preferences and increase policy acceptance.

## 1 INTRODUCTION

Unhealthy lifestyles are increasingly contributing to the global burden of disease. Noncommunicable diseases (NCDs) are a major public health challenge, and recent research by the World Health Organization (WHO) showed that over 80% of common NCDs, such as cardiovascular diseases, can be prevented by eliminating modifiable risk factors such as unhealthy lifestyle behaviours (e.g., smoking).^1^ This suggests that—at least some part of—current healthcare expenditures could potentially be saved by promoting individual responsibility for a healthy lifestyle.

Internationally, there are several initiatives to promote healthy lifestyles and reduce NCDs. The WHO Framework Convention on Tobacco Control is one of the most widely embraced treaties in United Nations (UN) history.^2^ This led to many initiatives, such as the ambition to have a tobacco‐free generation in the Netherlands by 2040.^3^, ^4^ Furthermore, the UN have set the goal to reduce premature mortality from NCDs by one‐third in 2030.^5^ Despite the increased interest in promoting healthy lifestyles, current healthcare expenditures continue to rise. Priority setting in healthcare is often subject to public and political debate. A recurring topic is the standpoint that resources allocated to the treatment of avoidable disease burden (e.g., burden caused by modifiable behaviour) could also be spent on interventions preventing or treating diseases that are not lifestyle related and, in relation, that individual responsibility for health could also be used as a rationing criterion.^6^, ^7^, ^8^

To allocate available healthcare resources in an equitable and efficient manner, many countries incorporated criteria into their decision‐making framework that relate to the necessity, effectiveness and cost‐effectiveness of the intervention and the feasibility of reimbursing it from public funding.^9^, ^10^ Box 1 shows the reimbursement criteria of the Netherlands.

Box 1 **Overview of reimbursement criteria used in the Netherlands^11^**

**Effectiveness**

How does treatment benefit a patient?

**Cost‐effectiveness**

Effects and all cost‐consequences of a (new) treatment will be set off against the treatment normally used up till that moment. Expressed in costs per QALY.

**Necessity (of care and of insurance)**

Is the disease burden serious?

Are the treatment cost too high for an individual to pay for?

**Feasibility**

Is inclusion of the (new) treatment in the basic healthcare package feasibile?

The increased prevalence of unhealthy lifestyles and their negative impact on health raise the question of whether it would be appropriate to consider individual responsibility for health as an additional criterion for rationing healthcare. Allocating responsibility to individuals for the health effects of their lifestyle, however, is controversial.^12^ There is no consensus on whether lifestyle choices can be considered as autonomous decisions, and an extensive body of evidence indicates that ill health is likely caused by multiple factors,^13^ both medical and nonmedical.^14^

Policies considering individual responsibility in the decision‐making framework for reimbursement of health interventions are scarce, but there are some. A local health committee in the United Kingdom announced a policy that postpones nonurgent surgery for people who smoke or are overweight until they reach a certain health level.^15^, ^16^ This policy aims ‘to support patients whose health is at risk from smoking or being very overweight’. In Germany, individual responsibility for health has relatively broad support as key elements from an important healthcare reform in 2007 involved the following: ‘insured persons may no longer claim‐free treatment for complications arising from certain “lifestyle choices”’.^17^ Policy proposals and debates about individual responsibility for health are more common, but consensus on its role in priority setting has not been reached. In Sweden, the responsibility principle was first rejected in 1995, but later in 2007, it was again promoted as a potential solution for the dilemmas in the current ethical platform.^18^ Considering that state responsibility is one of the main features of the welfare regimes of Scandinavian countries,^19^ this shift from collective state responsibility towards individual responsibility seems remarkable. In Norway, personal health responsibility has been repeatedly rejected as it seemingly challenges their core values of equality, inclusion and solidarity.^20^

In 2001, in the Netherlands, following the intense public debate about policy options to limit the rise in healthcare expenditures, the National Health Care Institute (ZIN) assessed the feasibility of implementing an additional decision‐making criterion related to individual responsibility for a healthy lifestyle.^6^ ZIN concluded that there were alternative policies in place (e.g., taxes) to compensate lifestyle‐related healthcare costs, and that a lifestyle criterion would likely not alter reimbursement decisions based on the four criteria currently included in the decision‐making framework (see Box 1). Despite the public debate potentially favouring a role for individual responsibility for health, this assessment was merely conducted on a theoretical level. An empirical study from 2010, investigating public preferences in 10 European countries on general principles for healthcare priority setting, found that taking individual responsibility for health was important in one of the five distinguished views;^21^ approximately 11% of the public in the Netherlands supported this particular view.^22^ This former study focused on viewpoints among the general population. The current study contributes by also including experts, enabling a direct comparison between their viewpoints and those of the public. In addition, the former study focused on the general principles regarding the healthcare priority setting, of which ‘individual responsibility’ was one. The current study examines more in depth the relative importance of a lifestyle criterion in the context of the decision‐making framework. Finally, in the 10 years since the previous study, the public debate about health lifestyle and own responsibility has continued; therefore, views on the relevance of a lifestyle criterion in healthcare decision‐making may have evolved.

To gain insight into the relative importance of individual responsibility for health relative to the currently applied reimbursement decision criteria, this study uses Q‐methodology to examine the viewpoints on this topic among the public and experts in healthcare in the Netherlands. The results of this study provide insight into shared viewpoints as well as the diversity of viewpoints regarding this topic. In addition, it will help identify if there are group(s) in society that potentially support or oppose incorporating a lifestyle criterion into priority setting, in addition to the current applied decision criteria.

## 2 METHODS

### 2.1 Participants

We collected data among experts (*n* = 37) and the public (*n* = 44) in June 2019. The sampling strategy in Q‐methodology can be compared to that of qualitative studies as the aim is to include data‐rich participants.^23^ Participants were recruited using convenience and snowball sampling methods to (1) obtain a varied, yet balanced sample of the public in terms of age, sex, educational level, political preferences and lifestyle (smoking status, alcohol consumption and body mass index [BMI]), recruited via different informal channels at sport facilities, in specific neighbourhoods and via personal connections, and (2) include a variety of experts, that is, Master‐ and PhD‐level students in health policy, policymakers, policy advisors and researchers in the healthcare sector. Participants were recruited from Erasmus University Rotterdam, Erasmus Medical Center, National Institute for Public Health and the Environment (RIVM), ZIN and the Ministry of Health, Welfare and Sport (VWS) in the Netherlands.

### 2.2 Q‐methodology

We applied Q‐methodology to identify public and expert viewpoints on the importance of using a lifestyle decision criterion relative to the currently applied decision criteria for the healthcare priority setting in the Netherlands. We conducted our study in three consecutive steps that are common to Q‐methodology studies,^23^ each further explained in the following paragraphs.

### 2.3 Statement set

To arrive at a comprehensive statement set reflecting current practice and debate on the healthcare priority setting, we used the decision criteria currently used by ZIN as domains to structure the statement set development (see Box 1). We supplemented this with a lifestyle criterion and a domain related to moral arguments that participants could deem relevant in this context.

To collect a set of statements that broadly covered our topic of interest, we reviewed the relevant literature, including previous Q‐methodology studies that focused on general principles for healthcare priority setting,^21^, ^24^ policy documents, research reports, news articles and social media. Based on this review, we identified over 100 statements on decision criteria for the healthcare priority setting. In multiple iterations, these statements were structured according to the six identified domains. After removing duplicate statements and rounds of editing to improve clarity and balance in the phrasing of the statements, we arrived at a selection of 34 statements, with each domain represented by 4–6 statements. Material S1 shows the statements per domain, together with their source of origin.

The comprehensiveness and wording of the statement set were assessed by a policymaker with expert knowledge about the reimbursement process in the Netherlands and by a researcher with expert knowledge on Q‐methodology. Finally, the statement set was pilot‐tested with five members of the public and six independent researchers. Based on these results, we made some minor changes to the wording of four statements to improve clarity. Considering that these changes were minor and did not alter the content of the statements, we merged the data collected in the pilot and main phases of the study for analysis.

### 2.4 Data collection

**Figure 1:**
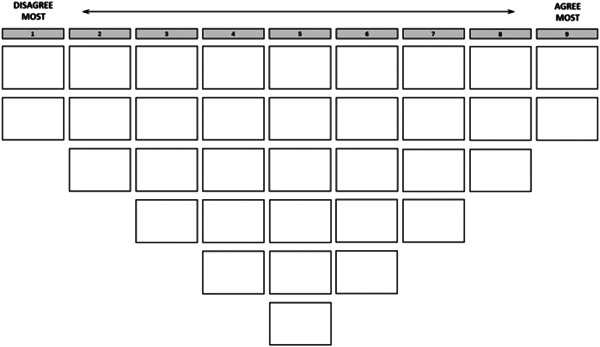
Ranking grid

We conducted the interviews, during which the participants ranked and subsequently explained their ranking of the statements, either at home (the public) or at work (experts). Each interview started with an introduction to the ranking exercise. Then, participants received the 34 cards with the printed statements, in a randomized order, and a ranking grid (see Figure 1). The participants were asked to carefully read each statement and allocate them to one of three piles that indicated whether they ‘agreed’, ‘disagreed’ or were ‘neutral’ to the statement. Participants were then instructed to rank the statements on the grid, starting with the pile of statements with which they ‘agreed’, followed by ‘disagreed’ on the left side, and finally by placing the statements in the ‘neutral’ pile. Once the statements were placed on the grid, participants were given time to reflect on their ranking and make some final changes. After completing the exercise, participants were asked to explain in writing why they placed certain statements at the extreme ends of the grid. Finally, participants completed a short questionnaire on their background characteristics, amongst which was their current lifestyle.

### 2.5 Data analysis

We subjected the data to a principal component analysis, followed by oblimin rotation, to gain insight into the viewpoints of experts and the public on the relative importance of a lifestyle decision criterion.

Different factor solutions were evaluated based on the following statistical properties: Eigenvalue of each factor >1, a low to moderate correlation between viewpoints (i.e., *ρ* < 0.50) and a minimum of two nonconfounded participants (i.e., exemplars) statistically significantly associated with each factor. In addition to these statistical properties, the interpretability of factors was evaluated by inspecting their coherence and distinctiveness.

For the selected factors, we computed factor arrays (i.e., weighted average ranking of the statements by exemplars), which represent how a participant that perfectly correlated with a factor, would rank the statements. These arrays were used for the interpretation and description of the factors as viewpoints on the relative importance of decision‐making criteria in healthcare decision‐making. The relative position of the statements in the array of a factor and statistically significant differences in position between factors were used to develop a narrative for each factor. Particular attention was paid to the statements that are characterizing for the factor, that is, those positioned on the extreme ends of the composite ranking, and the distinguishing statements for that factor, that is, those with a statistically significantly (*p* < .01) different position in the composite ranking of the factor as compared to the other factors. Finally, statements that did not differ statistically significantly in their position between any pair of factors were inspected. We used the qualitative data of the exemplars to verify and specify the interpretation of the factor. Exemplar quotes were used to illustrate the interpretation of the factors in the words of the participants. We used Rstudio 2.2.1335 and the qmethod package for analysing the data.^25^

### 2.6 Ethics

Before the study, participants received information about the objective and procedures of the study. All participants had the opportunity to ask questions and could withdraw if desired. Participants were assured that their data would be anonymized. Informed consent was provided by all participants before data collection. The ethical review board of the Erasmus School of Health Policy & Management assessed and waived approval for the study (20‐30 Dieteren).

## 3 RESULTS

### 3.1 Sample characteristics

The sample characteristics are presented in Table 1. The public sample was evenly distributed across age and sex; and most of the participants were highly educated. Of the experts, 81% were aged between 18 and 35 years, and about 50% were students who followed courses to obtain a Masters' degree at the university. Compared to general population statistics for the Netherlands in 2019, the participants more frequently had a healthy BMI (<25.0) and more often reported excessive alcohol consumption. Smoking was more prevalent among the group of experts, and excessive drinking was more prevalent among the group from the public.

**Table 1:** Sampling characteristics of the full sample of participants

Personal characteristics		Public (*n* = 44)	Experts (*n* = 37)	Dutch population statistics^a^
		% (*n*)	% (*n*)	%
Age				
18–35		36.4 (16)	81.1 (30)	22.6
36–55		34.1 (15)	13.5 (5)	26.7
55+		29.5 (13)	5.4 (2)	31.3
Gender				
Female		50.0 (22)	56.8 (21)	50.0
Male		50.0 (22)	43.2 (16)	50.0
Highest completed educational level				
Low		13.6 (6)	–	30.6
Medium		31.8 (14)	–	37.1
High		52.3 (23)	100 (37)	30.8
BMI				
≤24.9		65.9 (29)	89.2 (33)	50.5
25.0–29.9		31.8 (14)	2.7 (1)	34.8
≥30.0		2.3 (1)	5.4 (2)	14.7
Not stated		–	2.7 (1)	
Smoker				
Yes		13.6 (6)	22.0 (8)	21.7
No		59.1 (26)	65.0 (24)	45.7
Ex‐smoker		27.3 (12)	13.0 (5)	32.6
Excessive alcohol consumption^b^				
Yes		18.2 (8)	10.8 (4)	8.5
No		81.8 (36)	89.2 (33)	92.5
Expert type				
Policymaker		–	27.0 (10)	–
Researcher		–	32.5 (12)	–
Master's/PhD student		–	40.5 (15)	–

^a^ *Source*: Statistics Netherlands (https://www.cbs.nl).

^b^ Categorisation based on national guidelines (for women >14 glasses p/w, excessive for men >21 glasses p/w).

### 3.2 Factor analysis

A four‐factor solution was selected. The Eigenvalues of the factors were between 5.8 and 12.8, and 67 of the 81 participants that loaded statistically significantly on one factor. Table 2 shows the low to moderate correlation between the factors. Factors 1 and 3 show the highest correlation (*ρ* = 0.41), and Factors 3 and 4 show the lowest correlation (*ρ* = 0.23).

**Table 2:** Correlation between factors

	Factor 1	Factor 2	Factor 3	Factor 4
Factor 1	1	0.26	0.41	0.34
Factor 2	0.26	1	0.39	0.34
Factor 3	0.41	0.39	1	0.23
Factor 4	0.34	0.34	0.23	1

Table 3 shows the factor loadings of participants, ordered in terms of the study sample and statistical significance. The four factors were defined by 27, 22, 11 and 7 participants, respectively. Factors 1 and 4 both had one participant with a negative factor loading, and hence were interpreted as being bipolar. The explained variance was 47.2%.

**Table 3:** Participants' characteristics and factor association

ID	Study sample	Factor 1 (*n* = 27)	Factor 2 (*n* = 22)	Factor 3 (*n* = 11)	Factor 4 (*n* = 7)
**1**	Expert	**0.70**	−0.12	0.05	0.13
**2**	Expert	**0.75**	−0.23	−0.25	0.34
**3**	Expert	**0.39**	0.12	0.08	0.27
**4**	Expert	**0.56**	0.01	0.29	−0.09
**5**	Expert	**0.64**	0.49	−0.11	0.07
**6**	Expert	**0.77**	−0.06	0.21	−0.06
**7**	Expert	**0.52**	−0.15	0.18	0.37
**8**	Expert	**0.67**	−0.06	0.07	−0.32
**9**	Expert	**0.84**	−0.06	0.09	−0.06
**10**	Expert	**0.66**	−0.21	0.18	0.06
**11**	Expert	**0.58**	0.15	0.00	0.25
**12**	Expert	**0.70**	0.13	0.04	−0.11
**13**	Expert	**0.54**	0.17	−0.06	0.07
**14**	Expert	**0.66**	−0.32	0.31	0.16
**15**	Expert	**0.68**	0.01	0.06	0.26
**16**	Expert	**0.59**	0.30	−0.11	0.38
**17**	Expert	**0.61**	0.22	0.26	−0.08
**18**	Expert	**0.57**	0.33	0.13	0.02
**19**	Expert	**0.56**	0.04	0.21	−0.20
**20**	Expert	**0.51**	0.42	−0.02	0.24
**21**	Public	**−0.56**	0.22	0.35	0.32
**22**	Public	**0.60**	0.52	0.00	−0.23
**23**	Public	**0.49**	0.11	0.25	−0.09
**24**	Public	**0.46**	0.03	0.01	0.26
**25**	Public	**0.72**	−0.24	0.10	−0.09
**26**	Public	**0.64**	0.24	−0.26	0.25
**27**	Public	**0.50**	0.18	0.18	0.20
**28**	Expert	0.01	**0.59**	0.42	0.11
**29**	Expert	0.16	**0.66**	−0.15	0.00
**30**	Expert	0.05	**0.56**	−0.23	049
**31**	Expert	0.37	**0.60**	−0.07	0.23
**32**	Expert	0.04	**0.63**	0.01	0.37
**33**	Expert	0.10	**0.45**	0.31	0.16
**34**	Expert	0.31	**0.41**	0.09	0.17
**35**	Public	−0.21	**0.62**	−0.04	−0.12
**36**	Public	−0.12	**0.67**	0.07	−0.29
**37**	Public	−0.03	**0.72**	0.09	−0.16
**38**	Public	−0.09	**0.81**	−0.17	−0.11
**39**	Public	−0.01	**0.41**	−0.14	0.21
**40**	Public	−0.14	**0.76**	0.16	−0.02
**41**	Public	−0.04	**0.70**	0.21	0.10
**42**	Public	−0.07	**0.50**	0.37	−0.02
**43**	Public	0.06	**0.64**	0.25	0.37
**44**	Public	−0.04	**0.75**	−0.15	0.11
**45**	Public	0.00	**0.74**	0.02	0.03
**46**	Public	0.30	**0.52**	−0.08	0.36
**47**	Public	0.07	**0.50**	0.02	−0.21
**48**	Public	0.12	**0.72**	0.28	−0.29
**49**	Public	0.02	**0.59**	0.20	0.09
**50**	Expert	0.31	−008	**0.69**	−0.10
**51**	Expert	0.41	0.06	**0.53**	0.09
**52**	Expert	0.00	0.21	**0.56**	0.08
**53**	Public	0.16	−0.06	**0.82**	−0.04
**54**	Public	0.09	0.22	**0.59**	−0.12
**55**	Public	0.18	0.18	**0.65**	0.08
**56**	Public	0.14	0.26	**0.52**	0.31
**57**	Public	−0.08	0.15	**0.60**	−0.34
**58**	Public	0.04	0.09	**0.61**	−0.02
**59**	Public	0.23	0.25	**0.41**	0.09
**60**	Public	−0.09	−0.17	**0.62**	0.33
**61**	Expert	0.17	0.30	0.20	**0.41**
**62**	Expert	−0.01	−0.08	0.04	**0.82**
**63**	Public	−0.29	0.32	0.02	**0.54**
**64**	Public	−0.06	0.36	0.02	**−0.49**
**65**	Public	0.20	0.27	−0.18	**0.48**
**66**	Public	0.27	0.03	0.21	**0.55**
**67**	Public	0.15	0.23	−0.15	**0.44**
**68**	Public	−0.01	0.19	0.32	0.11
**69**	Public	0.34	0.24	0.42	0.12
**70**	Public	−0.36	0.47	0.39	0.04
**71**	Public	0.27	0.47	−0.04	0.45
**72**	Public	0.26	0.26	0.23	0.26
**73**	Public	0.14	0.16	−0.05	0.29
**74**	Public	−0.33	0.37	0.45	0.06
**75**	Expert	0.29	0.38	0.11	0.22
**76**	Expert	0.35	−0.03	0.3	0.22
**77**	Expert	0.43	0.03	0.48	0.26
**78**	Expert	0.31	0.28	0.36	0.03
**79**	Expert	0.34	0.12	0.17	0.27
**80**	Expert	0.48	−0.21	0.23	0.4
**81**	Expert	0.45	0.36	−0.46	0.21
**Explained variance (sum: 47.2%)**		**15.8%**	**14.6%**	**9.6%**	**7.2%**

The automatic flagging procedure in PQ method software was used to identify defining sorts (bold) according to the following rule: Flag loading a: if (1) *a* ^2^ > *h* ^2^/2 (factor ‘explains’ more than half of the common variance) and (2) *a* > 1.96/√(*N* items) (loading significant at *p* < .05).

In the following sections, each factor is described with reference to the positioning of statements in the factor array (see Table 4). Notation is in line with previous Q‐methodology studies,^24^ as # indicates the statement number, followed by the factor score of that statement. For instance, (#10 + 3) indicates that statement number 10 had a factor score of +3 in the respective factor array. When exemplar quotes are used in the descriptions of the viewpoints, the participant's identification number is used for reference.

**Table 4:** Statement set and factor arrays

#	Statements	Viewpoints^a^, ^b^			
		V1	V2	V3	V4
1	Access to healthcare should be based on medical need	**+3**	0	+1	**−4**
2	People with a severe condition should be treated with priority over people with a nonsevere condition	**+**1	0	**+**2	**+3**
3	A treatment for a nonsevere condition should not be reimbursed	*−2*	*−3*	*−2*	*−4*
4	If it is possible to save a life, every effort should be made to do so	−2	**+3**	−2	−1
5	If there is no alternative treatment available, the only available treatment must be reimbursed	**−4**	**+2**	**+**1	**+**1
6	Healthcare should focus on patients who need care the most	**+**2	**+**1	**+**1	**−1**
7	People can pay for inexpensive treatments out of pocket	0	**−1**	**+**1	***−3***
8	People with a higher income should co‐pay for care more often	0	**+**1	**+3**	**−3**
9	Copayment is acceptable to prevent excessive use of medication	**+2**	**0**	**+3**	**+1**
10	Patients should never have to pay themselves for treatment of a serious condition	*−1*	**+** *2*	*−1*	**+** *3*
11	The current basic benefits package should provide less coverage; more treatments should be included in the supplementary insurance policies	*−1*	*−1*	*−1*	*−1*
12	To ensure that patients will only use necessary care, patients can pay for the first treatments themselves	−1	**−2**	0	**−2**
13	Priority should be given to those treatments that generate the most health benefits	**+1**	**0**	**0**	**+4**
14	There is no point in including treatments in the basic benefits package that do not generate considerable health benefits	**+**2	**−1**	**0**	**+**2
15	Treatments that restore health to a level that is sufficient for participating in activities of daily living should be given priority	0	0	−1	**+** ***2***
16	There is no use in providing treatment when the result is still a very poor state of health	0	**−2**	−1	**+3**
17	The improvement in quality of life is the most important	**+3**	**+4**	**+**2	**+**1
18	A treatment should only be reimbursed if there is scientific proof that it is effective	**+**3	**0**	**−2**	**+**2
19	When having to choose between two treatments that cost the same, funding should be provided to the treatment that results in the biggest health gain	**+4**	**+**2	**+**2	**+4**
20	Treatments that are very costly in relation to their health benefits should not be reimbursed	**+1**	−2	**−3**	−1
21	If a treatment is very costly in relation to its health benefits, but is the only treatment available, it should still be reimbursed	**−4**	**+3**	0	0
22	If the total costs of treatment of a disease (for all patients) are high, this treatment should receive less priority	**−1**	−4	−4	**0**
23	Whether or not people have caused a disease themselves should not be relevant	0	**+2**	**−2**	0
24	Individual responsibility should not be taken into account because people do not always have control over their way of living	**+**1	**+**1	**−3**	**−1**
25	People who live a healthy life should be prioritized over people with an unhealthy lifestyle	**−3**	−2	**0**	−2
26	For treatments of diseases that are the result of lifestyle choices, payment of the treatment must also be an individual responsibility	*−2*	*−3*	*0*	*0*
27	It is more important to prevent ill health than it is to cure ill health once it occurs	**+** *1*	**+** *3*	**+** *4*	**+** *2*
28	If people become ill through no fault of their own, they should receive priority over people who are in some way responsible for their illness	*−3*	*−3*	**+** *1*	*0*
29	If there is a way of helping patients, it is morally wrong to deny them this treatment	−1	**+4**	−1	**0**
30	The government should not interfere with the lifestyle of individuals	**−3**	−1	**−3**	−2
31	Children's health should be given priority over adults' health	**0**	−1	**+2**	−2
32	If a lifestyle has negative consequences for others, intervention is acceptable	**+**2	**+**1	**+3**	**+**1
33	Poorer people should be given priority because they do not have the same opportunities in life	−2	−4	**−4**	−3
34	Everyone has a right to healthcare, but this does not mean that everything can always be reimbursed	**+4**	**+**1	**+4**	**+**1

^a^ Bold denotes the distinguishing statements.

^b^ Italic denotes the consensus statements.

### 3.3 Viewpoint 1: Access to cost‐effective treatments based on need

People with this view believe that everyone has a right to healthcare, but that this does not mean that everything can always be reimbursed (#34, +4). When a treatment is very costly in relation to its health benefits, even it is the only treatment available, it should not be reimbursed (#5, −4, #21, −4). Benefits in terms of quality of life improvements are most important (#17, +3; #14, +2; #13, +1). When choices need to be made between two treatments that cost the same, funding should be provided to the treatment that results in the biggest health gain (#19, +4).

*One should always choose for the best price‐quality ratio, more health gains for equal costs is always better. #ID 15*

People with this view believe that treatments should only be reimbursed if scientific evidence indicates that they are effective (#18, +3).

*To ensure solidarity within the [publicly financed healthcare] system, money should not be spent on treatments that don't work or are perhaps even harmful. # ID 2*

Access to healthcare should be based on patients’ need for care (#1, +3; #6, +2). Therefore, people who have a healthy lifestyle and those who fall ill through no fault of their own should not be prioritized over people with an unhealthy lifestyle and those who are in any way to blame for their disease (#25, −3; #28, −3). Neither should people be responsible for paying for the treatment of illnesses that result from their lifestyle choices (#26, −2). The ‘access based on need’ principle also implies that no particular weight is assigned towards prioritizing children over adults (#31, 0).

*Adults and children should be treated equally. Access to care should be based on the likelihood of successful treatment and the improvement in quality of life. # ID 16*

While people with this viewpoint believe that lifestyle should not play a role in reimbursement decisions, they do believe that the government holds some responsibility and government intervention is appropriate when people's lifestyle has negative consequences for others (#32, +2; #30, −3).

*The government has a responsibility to assist people in making an informed decision about their lifestyle behaviours. # ID 14*

### 3.4 Viewpoint 2: Life is precious and always worth saving

People with this view attach a high value to life and believe that prevention is important (#27, +3). When it is possible to save a life, every effort should be made to do so (#4, +3), and if there is a way of helping patients, it is morally wrong to deny them treatment (#29, +4). People with this view believe that quality‐of‐life gains are important (#17, +4), but treatment should be reimbursed even when patients' quality of life after treatment is still very poor (#16, −2) or when scientific proof on a treatment's effectiveness is limited (#18, 0). Of all viewpoints, this viewpoint is most opposed to *not* reimbursing treatment if they do not generate considerable health benefits (#14, −1). Even when a treatment is very costly in relation to its health benefits, but it is the only treatment available, it should still be reimbursed (#21, +3; #5, +2; #20, −2).

*We should do whatever it takes in order to make people healthy again. # ID 35*

*You never know for sure how someone will respond to treatment, thus deciding beforehand to not treat is not an option in my opinion… every life is worth saving. # ID 36*

People with this view believe that high total treatment costs (for all patients) (#22, −4) or low disease severity (#3, −3) should not affect reimbursement decisions.

*Costs should not play a role in reimbursement decisions. When there is any chance of improving someone's health, treatment should always be provided. # ID 45*

Factors like the cause of a disease (#23, +2; #28, −3; #25, −2) and the socioeconomic status (#33, −4) of patients are not considered relevant in reimbursement decisions.

### 3.5 Viewpoint 3: Prevention and individual responsibility for health

Like people with Viewpoint 1, people with this viewpoint believe that everyone has a right to healthcare, but that this does not mean that everything can always be reimbursed (#34, +4). Treatments that have high total costs (for all patients) should not receive less priority (#22, −4), nor should the costs of a treatment in relation to its health benefits be decisive for reimbursement (#20, −3). To prevent excessive use and avoid use of unnecessary healthcare, copayments are considered acceptable (#9, +3; #12, 0), in particular when this involves inexpensive treatments (#7, +1) and people who can easily afford this (#8, +3).

*For rich people, the costs are relatively lower and therefore they can support people with a lower socio‐economic status to overcome financial obstacles in healthcare. # ID 53*

People who hold this view believe that individual responsibility should play a role in reimbursement decisions (#24, −3) and in prioritizing healthcare (#23, −2), regardless of one's socioeconomic status (#33, −4).

*If you have a very unhealthy lifestyle and, therefore, need extra healthcare, it simply makes sense that your financial contribution to healthcare should be higher. # ID 57*

In addition, they value prevention as a means to keep the population healthy (#27, +4) and support government intervention when lifestyle choices have negative consequences for others (#32, +3; #30, −3).

*Society must not suffer from wrong choices made by others, especially when it comes to health. # ID 55*

Moreover, people with this viewpoint are of the opinion that one should bear responsibility for one's own lifestyle choices. Therefore, individual responsibility should play a role in reimbursement decisions (#24, −3) and in prioritizing healthcare (#23, −2).

*If you have a very unhealthy lifestyle and therefore need extra healthcare, it simply makes sense that your financial contribution to healthcare should be higher. #Participant 57*

This viewpoint is different from the other viewpoints by agreeing with the prioritisation of children over adults in healthcare (#31, +2) and by disagreeing with the condition that treatments should only be reimbursed when there is scientific proof on their effectiveness (#18, −2).

### 3.6 Viewpoint 4: Treatment outcome and cost‐effectiveness

People with this viewpoint believe that priority should be given to treatments that are effective (#13, +4; #14, +2), substantiated with scientific evidence (#18, +2), and also on their cost‐effectiveness (#19, +4).

*Even though it is difficult, and almost immoral to make a cost‐benefit analyses when there are human lives at stake, I do not think that we should avoid this. When a treatment does not lead to improved quality of life, it seems that people do avoid such decisions, and this tends to be a waste of money. # ID 65*

*In my opinion it does not make sense to choose for a treatment with less health gains but that has equal costs compared to another treatment. [Not reimbursing it] is a win‐win situation for both the patient and the government. # ID 61*

However, when there are no other treatments available, they tend to reimburse the only treatment that is available (#5, +1). People with this view believe that there is no use in providing treatment when the result is still a very poor state of health (#16, +3) and tend to disagree that, if it is possible to save a life, every effort should be made to do so (#4, −1). People with this view are least likely to believe that access to healthcare should be based on need for care (#1, −4). This view is also distinctive in terms of the belief that healthcare should not focus on patients who need care the most (#6, −1), nor that it would be morally wrong to deny treatment to patients (#29, 0). They deem it better to prioritize treatments that restore health to a level that is sufficient for participating in daily activities (#15, +2).

They believe that it is important to give priority to patients with a severe condition (#2, +3). However, they do not believe that treatments for non‐severe diseases should not be reimbursed (#3, −4), which aligns with their focus on treatment outcome and cost‐effectiveness. In this same context, they do not support individual responsibility for health (#25, −2; #24, −1) and paying for care out of pocket (#7, −3; #10, +3; 12, −2).

## 4 DISCUSSION

The aim of this study was to identify and describe viewpoints among experts and the public on applying a lifestyle decision criterion for the healthcare priority setting in the Netherlands relative to support for the currently applied decision criteria of necessity, effectiveness, cost‐effectiveness and feasibility. Our findings suggest that there are four viewpoints on this, each supported by a mix of experts and members of the public who participated in our study. The ‘Prevention and individual responsibility for health’ viewpoint seems to be supportive of the application of a lifestyle criterion. A notable finding was that this viewpoint was largely defined by the public sample, but that also some individuals of the expert sample were associated with this viewpoint. The majority of the experts participating in this study related more strongly to the ‘Access to cost‐effective treatments based on need’ viewpoint, which most closely reflects the current decision‐making framework in the Netherlands.

Three of the four viewpoints acknowledged the scarcity of resources and the necessity of priority setting based on, at least, some criteria. However, people with the viewpoint ‘Life is precious and always worth saving’ did not support rationing in healthcare, as they believed it to be morally wrong to deny treatment to patients. According to people with this view, life is priceless and always worth the effort made to save it. Patients should be offered treatment, even when the outcomes are likely to be very poor. People with this viewpoint and those with the viewpoint ‘Prevention and individual responsibility for health’ believe that healthcare costs should not play a major role in healthcare priority setting, and both attribute more importance to prevention as a means to keep the population healthy and healthcare costs low. The difference between these views is that the former associated this with the responsibility to adopt a healthy lifestyle, while the latter did not favour this. Viewpoints 1 and 4 both attribute importance to the cost‐effectiveness criterion; however, Viewpoint 4 favours reimbursement when there is only one treatment available, while Viewpoint 1 is not in favour of this. Moreover, Viewpoint 4 attaches more value to treatment outcomes, as they do not support the provision of treatment when the result is still a very poor state of health.

To a certain degree, the results of this study are in line with previous findings. A multicountry study reported that 50% of the public in the Netherlands would support smoking as a prioritizing criterion.^26^ A vignette study in the Netherlands showed that most participants were in favour of rewarding people with a healthy lifestyle instead of punishing those with an unhealthy lifestyle.^27^ In addition, two former Q studies on this topic also identified a positive attitude towards individual responsibility as a rationing criterion in one of the viewpoints.^21^, ^24^ Comparisons with these studies must be performed with caution. The objectives of the studies were slightly different, and therefore, also, the statement sets differed. The inclusion of experts in addition to members of the public also seems to have had a particular effect on our results since the viewpoint ‘Access to cost‐effective treatments based on need’ most closely reflects the current decision‐making framework in the Netherlands and was not identified in these former studies. Rogge and Kittel^26^ found that differences in attitude towards an individual responsibility criterion were best explained by rational choice theory, which suggests that people tend to prefer the distribution mechanism that is most advantageous for themselves.^28^ Our study approach was not suitable for confirming this. Traina and Feiring^29^ showed that clinicians were reluctant towards implementing a lifestyle criterion, mostly because they were concerned about the impact that such a principle could have on the most vulnerable people in society. Our study did not include clinicians as participants. Future research could extend on our study and examine how views of clinicians relate to those of experts and the public.

Several theories of justice (e.g., luck‐egalitarianism and libertarianism) favour consideration of individual responsibility as a mechanism to allocate scarce resources.^30^ However, feasibility issues seem to have a pivotal position in the discussion about the application of such a decision criterion. Most objections can be categorized into problems with (i) causality; (ii) efficiency; or (iii) universalisation. The causality problem refers to the multifactorial causes for many preventable diseases,^30^, ^31^ making it difficult to establish an unambiguous causal relation at the individual level between lifestyle choices and health outcomes. The efficiency problem claims that, although incorporating a lifestyle criterion should contribute to a more efficient allocation of scarce resources, the time and resources needed to determine patients' responsibility make it far from efficient.^32^, ^33^ Finally, the universalisation problem entails that applying a lifestyle criterion to its fullest means that many activities that pose health risks would need to be covered in such a mechanism, which is practically impossible.^34^ While these objections should be taken seriously, it does not mean that they eradicate the potential of a lifestyle criterion completely. If a lifestyle criterion is not introduced, one could argue that in the context of a collective health insurance system, citizens with a healthy lifestyle may be disadvantaged by citizens with an unhealthy lifestyle by claiming an ‘unfairly’ large share of the available healthcare resources for care that could perhaps have been prevented. Whether this free‐riding can be mitigated by introducing a lifestyle criterion needs to be further explored. An often mentioned objection is that the free‐riding argument assumes that there is a high degree of self‐control in lifestyle choices, while there is strong evidence that social determinants also matter.^35^, ^36^

### 4.1 Limitations

We acknowledge some limitations of our study. First, experts were aware that they were recruited because of their expertize. This knowledge might have led to social‐desirability or status‐quo bias, contributing to the identification of the viewpoint ‘Access to cost‐effective treatments based on need’ that closely resembles the current decision framework in the Netherlands. Moreover, the variety in experts was limited. Second, we based our statement set on existing materials from related studies that in some way addressed the relation between lifestyle and healthcare rationing. However, we did not conduct a systematic review of the underlying literature and, hence, aspects relevant to this relation may have been overlooked. We conducted a pilot study and obtained feedback on the comprehensiveness of the statement set from experts to verify whether aspects were missing. No missing aspects were identified, suggesting that it was representative of our topic of interest. Third, Factors 1 and 4 were bipolar, as both had one negative exemplar. Currently, there is no consensus on how to handle these exemplars in the analyses of the ranking data and interpretation of viewpoints. Some argue that negative exemplars should be excluded from the computation of the factor array as this would result in a more straightforward interpretation of the positive pole of the factor.^37^ Others, however, argue that they should be included as this results in a more balanced viewpoint that reflects the views of all participants who define it.^23^, ^38^ We followed the latter approach, but also inspected the solution without the negative exemplars as a robustness check and found that the viewpoints resulting from the two approaches did not differ significantly. Fourth, our results provide no insight into the prevalence of the viewpoints or into the strength of support for a lifestyle criterion, amongst larger samples of experts and the public in the Netherlands. Future research, in which the results of this study are integrated into a survey design,^22^ can shed light on this. Fifth, our data collection was finished before the COVID‐19 pandemic. Considering the burden of the pandemic on the healthcare system, viewpoints about criteria for healthcare rationing might have changed. Individual behaviours play an important role in the spread of viruses and the course of an infection; thus, the relevance of lifestyle and responsibility may also have changed in the meantime.

## 5 CONCLUSION

This study confirms findings from some previous studies indicating (some) support for a lifestyle criterion in the healthcare priority setting, but we also found viewpoints indicating clear objection to such a criterion. Further research using survey methods is needed to understand the extent of the controversy around this topic better. We anticipate that the role of individual responsibility in health(care) will remain a controversial topic of debate. Accounting for heterogeneity in policies aimed at addressing responsibility in healthcare seems pivotal to increase the likelihood of policy acceptance.

## CONFLICT OF INTERESTS

The authors declare that there are no conflict of interests.

## AUTHOR CONTRIBUTIONS

Charlotte M. Dieteren: *Conceptualisation, partial data collection, data curation, formal analysis, interpretation results, visualisation, writing – original draft*. Vivian T. Reckers‐Droog: *Conceptualisation, data curation, interpretation results, writing – review & editing*. Sara Schrama: *Conceptualisation, data collection, review*. Dynothra de Boer: *Conceptualisation, data collection, review*. Job van Exel: *Conceptualisation, investigation, interpretation results, supervision, writing – review & editing*. Members of the public were involved in the data collection of this study.

## Supporting information

Table S1. Original and used statements in the study with the source of origin and categorized by domain.

## Data Availability

Data are available upon request and placed in university repository.
