# Supplementary material for: Viewpoints among experts and the public in the Netherlands on including a lifestyle criterion in the healthcare priority setting
Source: Health Expect. 2021 Nov 29;25(1):333–44. doi: 10.1111/hex.13385 (PMC8849370; doi:10.1111/hex.13385)
Supplement: Supplementary file 1 — Table S1. Original and used statements in the study with the source of origin and categorized by domain. [file HEX-25-333-s001.docx]

# Supplementary file

Table S1. Original and used statements in the study with the source of origin and categorised by domain

| **Decision Criteria** | **Reference** | **Original statements** | **Used statements (ENG)** |
| --- | --- | --- | --- |
| Necessity of care | (Wouters et al., 2017) | Access to health care should be based on need for care, not on patient characteristics, such as their gender, age or ethnicity | Access to healthcare should be based on medical need. |
|  | (Wouters et al., 2017) | People with a severe condition should be treated with priority over people with a non-severe condition | People with a severe condition should be treated with priority over people with a non-severe condition. |
|  | (Zorginstituut Nederland, 2017) | Een interventie voor een aandoening met een hoge ziektelast moet wel vergoed worden/een interventie voor een aandoening met een lage ziektelast moet niet vergoed worden | A treatment for a non-severe condition should not be reimbursed. |
|  | (Wouters et al., 2017) | I fit is possible to save a life, every effort should be made to do so | If it is possible to save a life, every effort should be made to do so. |
|  | (Reckers-Droog et al., 2020) | Als er geen alternatieve behandeling beschikbaar is voor een aandoening, dan kan dit een reden zijn om de enige beschikbare behandeling te vergoeden. | If there is no alternative treatment available, the only available treatment must be reimbursed. |
|  | (Reckers-Droog et al., 2020) | De gezondheidszorg moet zich richten op die patiënten die de zorg het hardst nodig hebben. | Healthcare should focus on patients who need care the most. |
| Necessity of reimbursement | (Reckers-Droog et al., 2018) | People can pay for inexpensive treatment out of pocket | People can pay for inexpensive treatments out of pocket. |
|  | (Reckers-Droog et al., 2020) | Vermogende patiënten zouden vaker een eigen bijdrage kunnen betalen | People with a higher income should co-pay for care more often |
|  | (Reckers-Droog et al., 2020) | Om misbruik van medicijnen te voorkomen is in sommige gevallen een eigen bijdrage nodig | Co-payment is acceptable to prevent excessive use of medication |
|  | (Zorginstituut Nederland, 2017) | Patiënten zouden een dure behandeling nooit zelf hoeven te betalen | Patients should never have to pay themselves for treatment of a serious condition |
|  | (Reckers-Droog et al., 2020) | Wanneer behandelingen vanuit de aanvullende verzekering kunnen worden vergoed, hoeft deze niet in het basispakket te worden opgenomen | The current basic benefits package should provide less coverage, more treatments should be included in the supplementary insurance policies |
|  | (Reckers-Droog et al., 2020) | Om er voor te zorgen dat patiënten alleen gebruik maken van hulp die echt noodzakelijk is en om ervoor te zorgen dat zij echt beter worden, kunnen patiënten de eerste behandelingen zelf betalen | To ensure that patients will only use necessary care, patients can pay for the first treatments themselves. |
| Effectiveness | (van Exel et al., 2015) | Priority should be given to those treatments that generate the most health. | Priority should be given to those treatments that generate the most health. |
|  | (van Exel et al., 2015) | There is no point in providing treatments that do not generate considerable health benefits | There is no point in including treatments in the basic benefits package that do not generate considerable health benefits. |
|  | (Reckers-Droog et al., 2020) | Voorkeur moet uitgaan naar het herstellen van gezondheid tot een niveau dat voldoende is voor mensen om aan hun gebruikelijke activiteiten deel te kunnen nemen. | Treatments that restore health to a level that is sufficient for participating in activities of daily living should be given priority. |
|  | (Wouters et al., 2017) | There is no use in providing treatment when the result is still a very poor state of health. | There is no use in providing treatment when the result is still a very poor state of health. |
|  | (Baker et al., 2014) | The amount of health and quality of life improvement is the most important. It is about getting the greatest benefit for the most people | The improvement in quality of life is the most important. |
|  | (Zorginstituut Nederland, 2014) | Een behandeling moet alleen worden vergoed als het wetenschappelijk bewezen effectief is (‘evidence-based medicine’) | A treatment should only be reimbursed if there is scientific proof that it is effective. |
| Efficiency | Van Exel et al., (2015) | When having to choose between two treatments that both cost the same, funding should be given to the treatment that results in the biggest health gain. | When having to choose between two treatments that both cost the same, funding should be given to the treatment that results in the biggest health gain. |
|  | (Reckers-Droog., 2018). | Treatments that are very costly in relation to their health benefits should be withheld | Treatments that are very costly in relation to their health benefits should not be reimbursed |
|  | Wouters et al., (2017) | If a treatment is costly in relation to its health benefits, but the only treatment available, it should still be provided. | If a treatment is very costly in relation to its health benefits, but it is the only treatment available, it should still be reimbursed. |
|  | Reckers-Droog et al., (2018) | treatments that are very costly in relation to their health benefits should be withheld | If the total costs of treatment of a disease (for all patients) are high, this treatment should receive less priority. |
| Lifestyle | Baker et al., (2014) | Whether or not people have caused the illness themselves should not be relevant. If someone has got an illness through smoking, they are just as worthy of treatment as someone else. | Whether or not people have caused a disease themselves should not be relevant. |
|  | Wouters et al., (2017) | Individual responsibility Lifestyle should not be taken into account because people don’t always have control over their way of living. | Individual responsibility should not be taken into account, because people do not always have control over their way of living. |
|  | Wouters et al., (2017) | People who live a healthy life should be prioritized over people with an unhealthy lifestyle. | People who live a healthy life should be prioritized over people with an unhealthy lifestyle. |
|  | (Raad voor de Volksgezondheid en Zorg, 2014) | Voor behandelingen die het gevolg zijn van eigen keuzes in de leefstijl, moet de betaling ook eigen verantwoordelijkheid zijn. | For treatments of diseases that are the result of lifestyle choices, payment of the treatment must also be an individual responsibility. |
|  | Wouters et al., (2017) | It is more important to prevent ill health than it is to cure ill health once it occurs. | It is more important to prevent ill health than it is to cure ill health once it occurs. |
|  | (Reckers-Droog et al., 2020) | Mensen die buiten hun schuld om een ziekte hebben gekregen moeten voorrang krijgen op mensen die zelf verantwoordelijk zijn voor hun ziekte. | If people become ill through no fault of their own, they should get priority over people who are in some way to culpable for their illness. |
| Moral principles | Wouters et al., (2017) | If there is a way of helping patients, it is morally wrong to deny them this treatment. | If there is a way of helping patients, it is morally wrong to deny them this treatment. |
|  | (Raad voor de Volksgezondheid en Zorg, 2014) | Leefstijlbeïnvloedingen zijn altijd onacceptabel omdat deze interfereert met de individuele vrijheid | Government should not interfere with the lifestyle of individuals. |
|  | Wouters e al., (2017) | Children’s health should be given priority over adults’ health. | Children’s health should be given priority over adults’ health. |
|  | (Raad voor de Volksgezondheid en Zorg, 2014) | Als je leefstijl geen negatieve gevolgen voor andere heeft, dan is beïnvloeding ervan acceptabel | If a lifestyle has negative consequences for others, intervention is acceptable. |
|  | Wouters et al., (2017) | Poorer people should be given priority because they don’t have the same opportunities in life. | Poorer people should be given priority because they don’t have the same opportunities in life. |
|  | Wouters et al., (2017) | Everyone has a right to healthcare, but this does not extend beyond a certain basic level. | Everyone has a right to healthcare, but this does not mean that everything can always be reimbursed. |
